# A Retrospective Observational Study to Evaluate Adjacent Segmental Degenerative Change with the Dynesys-Transition-Optima Instrumentation System

**DOI:** 10.3390/jcm13020582

**Published:** 2024-01-19

**Authors:** Chi-Ruei Li, Shih-Hao Chen, Wen-Hsien Chen, Hsi-Kai Tsou, Chung-Yuh Tzeng, Tse-Yu Chen, Mao-Shih Lin

**Affiliations:** 1 Department of Neurosurgery, Neurological Institute, Taichung Veterans General Hospital, Taichung 407, Taiwan; fantastic1694@gmail.com (C.-R.L.); ken70218@gmail.com (M.-S.L.); 2 Department of Orthopaedics, Tzuchi General Hospital, Taichung 427, Taiwan; shihhaotzuchi@gmail.com; 3 Department of Radiology, Taichung Veterans General Hospital, Taichung 407, Taiwan; chenws.tw@gmail.com; 4 Functional Neurosurgery Division, Neurological Institute, Taichung Veterans General Hospital, Taichung 407, Taiwan; 5 Department of Rehabilitation, Jen-Teh Junior College of Medicine, Nursing and Management, Miaoli 356, Taiwan; 6 Department of Orthopedics, Taichung Veterans General Hospital, Taichung 407, Taiwan; tcy0545@gmail.com

**Keywords:** adjacent segment disease, dynamic stabilization, Dynesys-Transition-Optima, lumbar vertebrae, prostheses and implants, spinal fusion, spinal stenosis, spondylolisthesis

## Abstract

Background: This study evaluates the impact of hybrid dynamic stabilization using the Dynesys-Transition-Optima (DTO) system on adjacent segment disease (ASD) in lumbar spinal stenosis patients with spondylolisthesis. Methods: From 2012 to 2020, 115 patients underwent DTO stabilization at a single center by a single neurosurgeon. After exclusions for lack of specific stabilization and incomplete data, 31 patients were analyzed. Follow-up was conducted at 6, 12, and 24 months postoperatively, assessing disc height, listhesis distance, and angular motion changes at L2–L3, L3–L4, and L5–S1. Results: L3–L4 segment (the index level), demonstrated a delayed increase in listhesis distance, contrasting with earlier changes in other segments. At two years, L3–L4 exhibited less increase in listhesis distance and less disc height reduction compared to L2–L3 and L5–S1. Notably, the L3–L4 segment showed a significant reduction in angular motion change over two years. Conclusions: In conclusion, while ASD was not significantly prevented, the study indicates minor and delayed degeneration at the index level. The L3–L4 segment experienced reduced angular change in motion, suggesting a potential benefit of DTO in stabilizing this specific segment.

## 1. Introduction

Musculoskeletal disorders, impacting approximately 1.71 billion individuals worldwide, are a predominant cause of disability. These conditions primarily involve impairments in muscles, bones, joints, and connective tissues [1]. The most frequently reported symptom across these disorders is pain, which varies from acute to chronic. A notable aspect of these disorders is lumbar spinal stenosis, with moderate cases presenting a prevalence of 21–30% and severe cases 6–7% [2]. This condition typically arises from degenerative changes, including disc height reduction, bone spur formation, thickening of the ligamentum flavum, and narrowing of the central spinal canal and lateral recesses. Spondylolisthesis, characterized by one vertebra slipping over another, further contributes to lumbar spinal stenosis, leading to a diminished space for spinal nerves and resultant symptoms.

In the diagnosis and understanding of degenerative spinal conditions, a plethora of analytical tools are utilized to investigate the pathogenesis [3,4,5]. Various intervention strategies are employed to manage the signs and symptoms of musculoskeletal conditions. These include patient education, rehabilitation, pharmacological treatments, and non-invasive techniques [6,7]. In cases where these methods prove insufficient, surgery is considered as the ultimate intervention for addressing pathological spinal conditions.

In recent decades, spinal fusion has been widely used for pathological spinal conditions. Even in such advanced lumbar interbody fusion techniques as conventional posterior lumbar interbody fusion (PLIF)/transforaminal lumbar interbody fusion (TLIF), second or revision surgery rates may still be as high as 36% [8]. Literature reviews have revealed that spinal fusions may increase the stress on the non-operated adjacent lumbar segments, causing the common complication of adjacent segmental degeneration over the long term [9,10]. Clinically, adjacent segmental disease (ASD) is problematic because it may necessitate further surgical management and risk adverse effects on daily functional outcomes [11]. The risk factors for surgery-related adverse effects include age, female gender, obesity, pre-existing degeneration, number of segments fused, and fusion procedure methods [12,13,14,15,16].

Biomechanically, rigid instrumentation may risk the degenerative alteration of adjacent level segments, especially cranial ones [17,18]. In a detailed biomechanical study, Lee et al. [19] found that higher intradiscal pressure within the adjacent levels was strongly associated with ASD. However, the greatest concerns in spine surgery are the risk factors for the onset of ASD and the choice of technique (i.e., fusion or non-fusion). Motion-preserving techniques might represent a feasible strategy to diminish the interruption of spinal column balance. Thus, dynamic instrumentation implants, which reduce biomechanical stress on the level adjacent to the instrumented segment and decrease the risk for ASD, Refs. [15,19,20] represent a practicable answer. One example is the Dynesys-Transition-Optima (DTO) system (Zimmer Spine Inc., Denver, CO, USA), which affiliates the dynamic stabilization of the cranial segment to the rigid instrumented level. Baioni et al. [21] showed that hybrid posterior lumbar fixation presented satisfying clinical outcomes in the treatment of degenerative disease at a 5-year follow up. In contrast, Herren et al. [22] found that dynamic instrumentation did not significantly ameliorate the development of ASD compared with the established rigid fusion procedure.

Concerning the association between dynamic instrumentation and ASD, studies have varied vastly in outcome. We posited that the variation in surgical indication and the discrepancy between one-level and multiple-level surgery may explain some of this variance. To reduce potential confounders, our study strictly focused on L4–L5 one-level fusion with cranially dynamic instrumentation. To explore adjacent segmental degenerative changes after DTO system instrumentation, this study centered on radiological changes after lumbar instrumentation as indicators of ASD in patients with at least a 2-year follow up.

## 2. Material and Methods

### 2.1. Patient Selection

**Figure 1:**
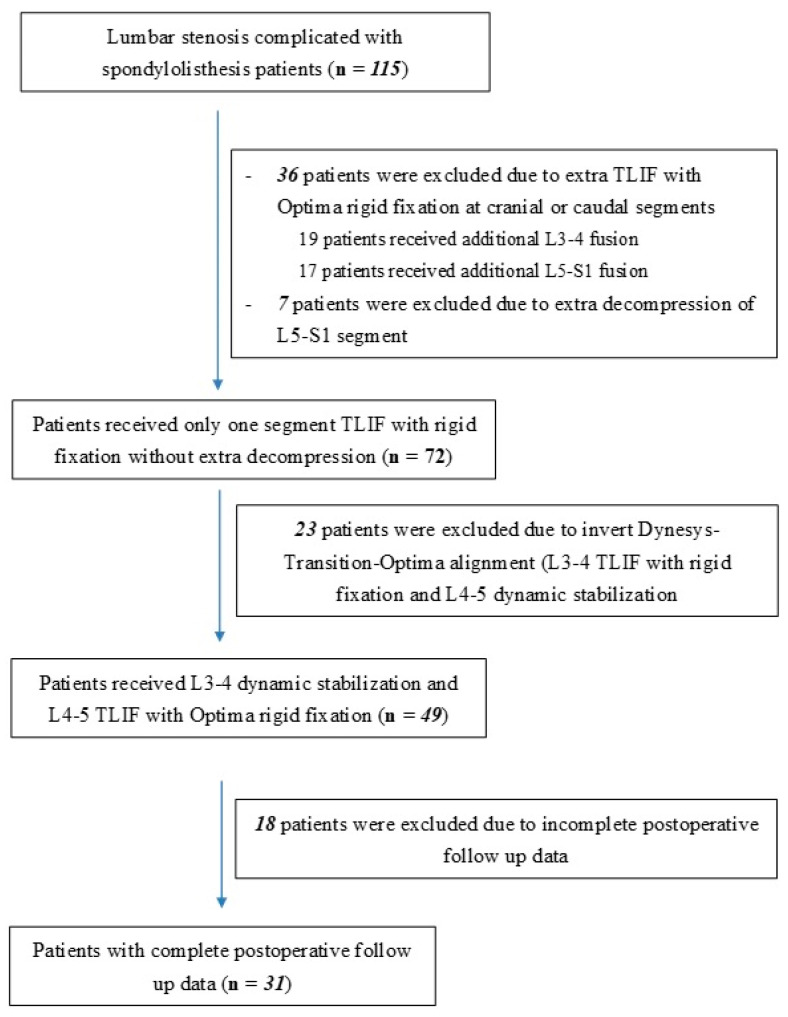
Flow diagram of inclusion and exclusion process.

In order to evaluate the impact of hybrid dynamic stabilization using the DTO system on ASD in lumbar spinal stenosis patients with spondylolisthesis, the present study recruited 31 patients who received hybrid dynamic pedicle screw insertion using the DTO system at a single medical center from a single neurosurgeon from 2012 to 2020 who also had documented radiological evaluation. Demographic data for these patients are shown in Table 1. The inclusion and exclusion process is shown in Figure 1. Follow up was conducted at 6, 12, and 24 months postoperatively, assessing disc height, listhesis distance, and angular motion changes at L2–L3, L3–L4, and L5–S1. This study was approved on 1 November 2022 by Taichung Veterans General Hospital Institutional Review Board (No. 19-12345) according to the Declaration of Helsinki. All subjects provided written informed consent to participate.

### 2.2. Radiological Evaluation

**Figure 2:**
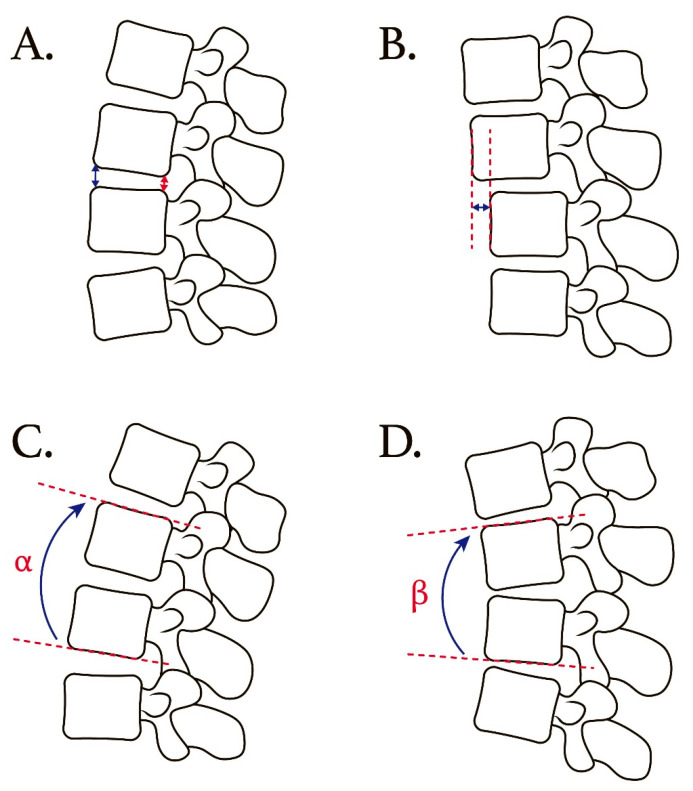
Schematic illustration showing radiological measurements of preoperative and postoperative lateral radiograms. (**A**) For disc height measurement, anterior disc height (blue double headed arrow) and posterior disc height (red double headed arrow) were measured. (**B**) Listhesis distance is defined as the distance between the posterior–lower portion of the upper vertebral body and the posterior border of the lower vertebral body. (**C**,**D**) Schematic drawing of angular motion change measure in extension/flexion view. Difference between α and β represents motion angular change.

Radiological imaging of the lumbar spine was performed (standard lumbar spine radiographs in the dynamic lateral views, including in the flexion and extension positions (Figure 2)). The radiological data were obtained at the preoperative visit, the day after the operation, and at approximately 6, 12, and 24 months postoperatively. Assessment of the adjacent segment included disc height, listhesis distance, and angular change while in motion. Signs of implant failure and screw loosening were also documented. All radiological assessments were applied at the L2–L3, L3–L4, and L5–S1 segments separately and reviewed independently by two neurosurgeons and one neuro-radiologist.

The anterior disc height (ADH) and posterior disc height (PDH) were measured on the lateral radiographs. The ADH was defined as the distance between the anterior border of the endplates in the consecutive vertebral body. Similarly, the PDH was defined as the distance between the posterior border of the endplates in the consecutive vertebral body. We used the average of ADH and PDH to represent the disc height value in this study. The listhesis distance was defined as the translation distance between the posterior–lower portion of the upper vertebral body and the posterior border of the lower vertebral body. These radiological parameters were measured at the L2–L3, L3–L4, and L5–S1 levels separately.

For the L2–L3 and L3–L4 levels, angular motion change at the adjacent segment was measured between the inferior endplate line of the upper vertebral body and the superior endplate line of the lower vertebral body on flexion/extension lateral radiographs. For the L5–S1 level, angular motion change at the adjacent segment was measured between the inferior endplate line of the L5 vertebral body and the superior endplate line of the S1 vertebral body on flexion/extension lateral radiographs.

### 2.3. Surgical Techniques

**Figure 3:**
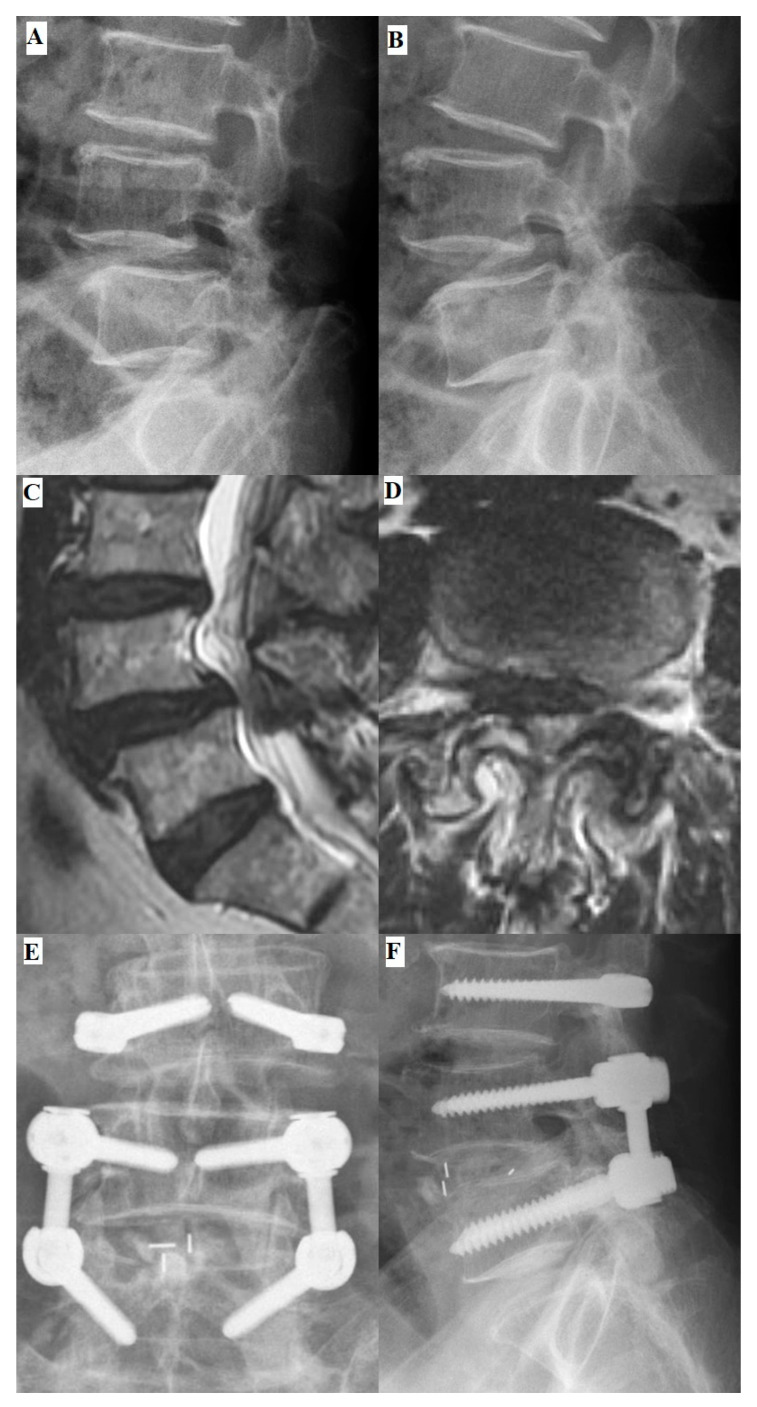
Preoperative and postoperative images of a 71-year-old male patient. The preoperative L-spine flexion (**A**) and extension (**B**) radiographs revealed L4–L5 segment spondylolisthesis and the formation of multiple osteophytes. (**C**) Preoperative magnetic resonance imaging (MRI) T2-weighted image sagittal view showed herniation of the intervertebral disc at the L4–L5 segment. (**D**) The axial preoperative MRI T2-weighted image revealed severe spinal stenosis with facet hypertrophy at the L4–L5 segment. Postoperative anterior–poster (AP) view (**E**) and lateral view (**F**) images revealed well-instrumented implantations and promising correction of the spondylolisthesis.

All patients received hybrid dynamic pedicle screw insertion using the DTO system with bilateral L3–L4 and L4–L5 laminotomies and L4–L5 interbody fusion (Figure 3). The Optima rigid part was instrumented between the L4 and L5 vertebrae and the Dynesys dynamic stabilization part placed cranially to the rigid part. The operation was performed via a posterior midline incision with the patient in a prone position under general anesthesia. The osteophytes, hypertrophic ligamentum flavum, and bulging disc were completely removed to ensure that the bilateral neural foramina and lateral recesses were well decompressed. To prevent postoperative spinal instability, bilateral facet joints, supra-spinous ligaments, and spinous processes were all carefully preserved, especially in the segments receiving Dynesys dynamic stabilization.

### 2.4. Statistical Analysis

All data analyses were performed with SPSS v23 statistical software (IBM Corp., Armonk, NY, USA). Kruskal–Wallis tests and Friedman tests were applied to determine if there were statistically significant differences between the L2–L3, L3–L4, and L5–S1 segments. All continuous variables were presented as the mean ± standard deviation. A *p*-value of 0.05 was set to be statistically significant.

## 3. Results

### 3.1. Demographic Data

The mean age of these 31 patients at surgery was 68.5 ± 7.5 years. For gender, the study group had 20 female patients and 11 male patients. No patient received revision surgery during the follow-up period.

### 3.2. Postoperative Segment Changes

For the L2–L3 segment, the preoperative mean disc height (average of the anterior disc height + posterior disc height) differed significantly from the 2-year postoperative value (0.74 ± 0.22 cm versus 0.48 ± 0.20 cm, *p* < 0.01). A significant increase in the listhesis distance between the preoperative and the 2-year postoperative data was also noted (0.24 ± 0.09 cm versus 0.51 ± 0.12 cm, *p* < 0.01).

A similar change in the L3–L4 segment was found. The preoperative mean disc height differed significantly from the 2-year postoperative value (0.75 ± 0.26 cm versus 0.52 ± 0.20 cm, *p* < 0.01). The preoperative and 2-year postoperative listhesis distance also differed significantly (0.25 ± 0.09 cm versus 0.43 ± 0.09 cm, *p* < 0.01).

For the L5–S1 segment, the preoperative mean disc height differed significantly from the 2-year postoperative value (0.85 ± 0.28 cm versus 0.56 ± 0.21 cm, *p* < 0.01). An increase in listhesis distance between the preoperative and 2-year postoperative data was also noted (0.28 ± 0.08 cm vs. 0.48 ± 0.12 cm, *p* < 0.01). The detailed data are shown in Table 2.

### 3.3. Disc Height Reduction

**Figure 4:**
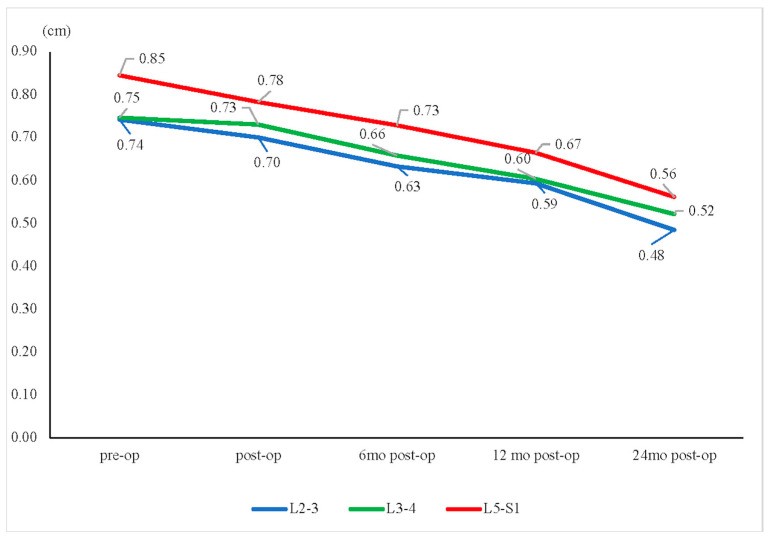
Disc height change. The mean disc heights for the L2–L3, L3–L4, and L5–S1 segments all showed continuous decreases over time, from preoperative (pre-op) to 24 months postoperative (post-op). * *p* < 0.05.

**Figure 5:**
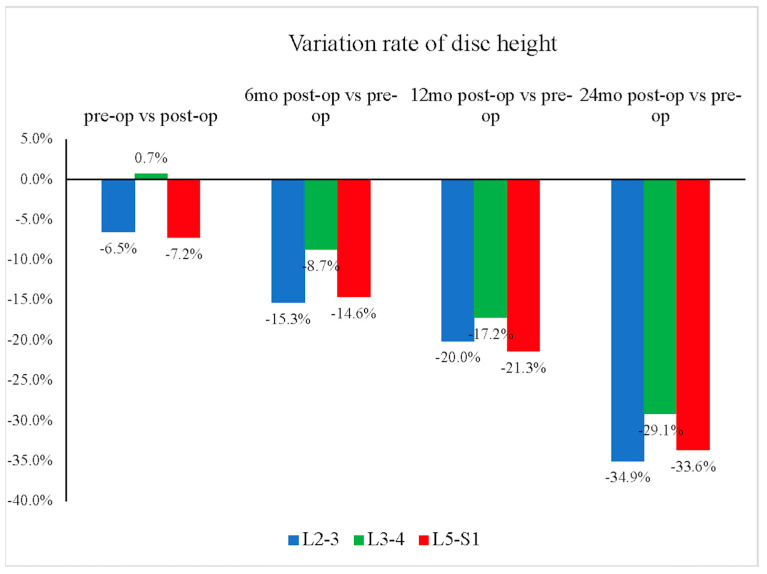
Reduction rate over time of average disc height by segment. The L3–L4 segment showed relatively less disc height reduction over time, from preoperative (pre-op) to 24 months postoperative (post-op), compared with the other groups (at 2 years, L3–L4: −29%; L2–L3: −35%; L5–S1: −34%, *p* = 0.549).

The mean disc heights for the L2–L3, L3–L4, and L5–S1 segments all showed continuous decreases (Figure 4). The L3–L4 segment showed relatively less disc height reduction than the other groups (L2–L3: −35%; L3–L4: −29%; L5–S1: −34%, *p* = 0.549) (Figure 5). Furthermore, our study showed that the L3–L4 segment took longer than other segments to show a significant reduction in disc height. For the L3–L4 segment, significant disc height reduction appeared 1 year after the operation; however, the alteration in other segments was shown earlier, at merely 6 months after the surgery.

### 3.4. Listhesis Distance

**Figure 6:**
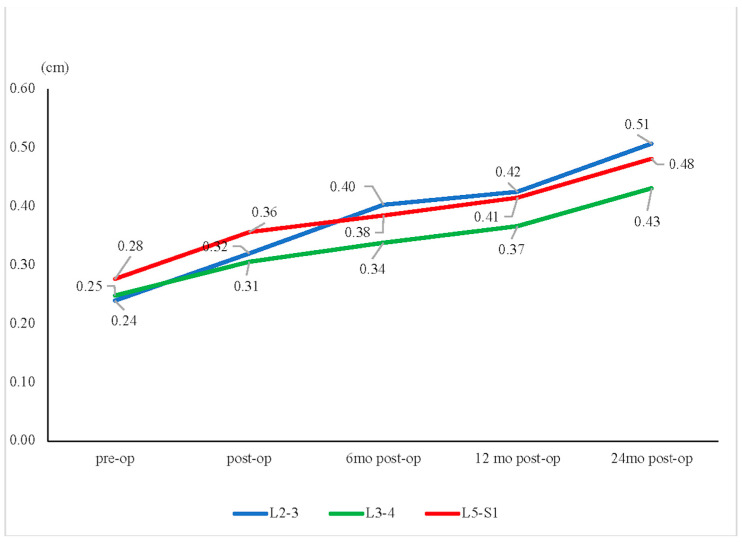
Listhesis distance change. In the listhesis distance, the L3–L4 segment showed a significantly lower increase over time, from preoperative (pre-op) to 24 months postoperative (post-op), compared with the L2–L3 and L5–S1 segments (at 2 years, L3–L4: 0.18 cm; L2–L3: 0.27 cm; L5–S1: 0.20 cm, *p* = 0.001).

For the 2-year change in listhesis distance, the L3–L4 segment showed a significantly lower increase than the L2–L3 and L5–S1 segments (L3–L4: 0.18 cm; L2–L3: 0.27 cm; L5–S1: 0.20 cm, *p* = 0.001) (Figure 6).

### 3.5. Motion Angular Change

**Figure 7:**
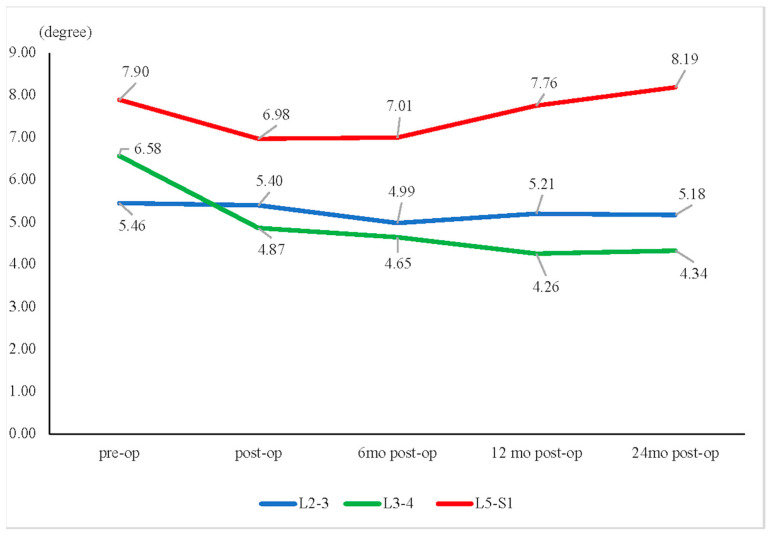
Motion angular change. * Only the L3–L4 segment revealed a significant decrease in motion angular change between the preoperative (pre-op) and 2-year postoperative (post-op) data (6.58° ± 3.78° pre-op vs. 4.34° ± 3.29° 24 months post-op, *p* = 0.023).

In the assessment of angular change between flexion and extension, only the L3–L4 segment showed a significant decrease from the preoperative data at 2 years (6.58° ± 3.78° versus 4.34° ± 3.29°, *p* = 0.023) (Figure 7).

## 4. Discussion

To evaluate the impact of hybrid dynamic stabilization using the DTO system on ASD in lumbar spinal stenosis patients with spondylolisthesis, the present study analyzed 31 patients from 2012 to 2020. The results showed that, at the L3–L4 segment (the index level), listhesis distance increased later than in other segments. At the two-year follow up, the L3–L4 segment exhibited a lower increase in listhesis distance and less disc height reduction, compared to L2–L3 and L5–S1.

**Figure 8:**
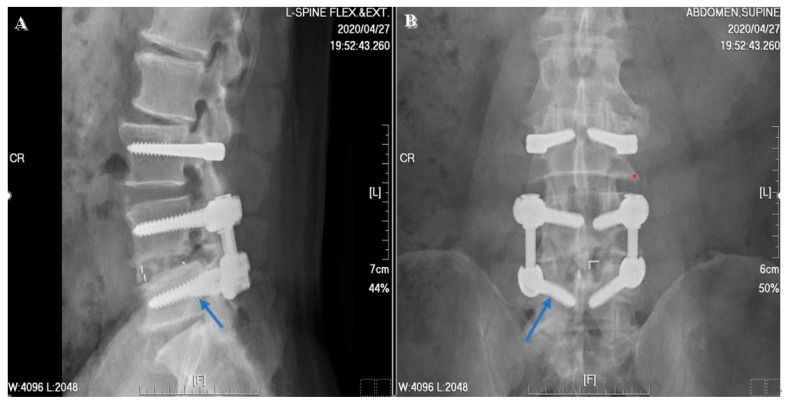
A 58-year-old male patient suffered from right L5 screw breakage. The 2-year postoperative follow-up image revealed right-side L5 screw breakage (blue arrow). (**A**) Lateral view. (**B**) AP view.

**Figure 9:**
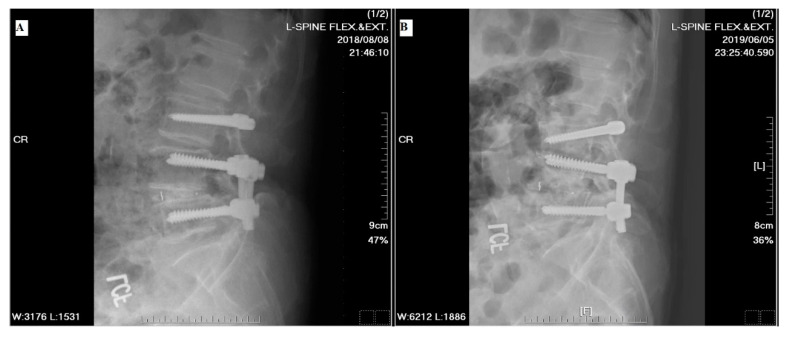
A 76-year-old male suffered from trauma-related implant loosening. (**A**) The 18-month postoperative radiographic follow up indicated the appropriate position of instrumentation. (**B**) The 24-month postoperative radiographic follow up revealed deviation in the bilateral L3, L4, and L5 screws after an automobile traffic accident.

Regarding the clinical outcomes associated with DTO instrumentation, neither Maserati et al. [20] nor Baioni et al. [21] reported any cases of implant-associated failure during a maximum follow up of 5 years. In contrast, Herren et al. [22] found an implant-dependent failure rate of 21.43% (*n* = 3) associated with the topping-off procedure. A relatively high rate of failure was also reported by Putzier et al. [23], who found both screw breakage (*n* = 2, 9.09%) and longitudinal rod breakage (*n* = 1, 4.55%) during a 6-year follow up in a prospective clinical trial of the Allospine Dynesys Transition System (Zimmer Biomet, GmbH, Winterthur, Switzerland), the prototype of the DTO. In our study, only one implant failure was noted at the 2-year postoperative follow up (Figure 8). Right L5 screw breakage was noted in the follow-up radiographs without any symptomatic problems. In addition, one patient excluded from the final data analysis, due to incomplete imaging follow up, suffered from implant loosening related to an automobile accident before his 2-year postoperative follow up. According to this patient’s radiographs, screws of the L3, L4, and L5 segments showed loosening signs (Figure 9). We checked the bone mineral density of this patient before surgery but found no evidence of osteoporosis.

To further examine implant failure in the DTO device, we reviewed the literature focused on finite element analysis during dynamic performance [24]. Theoretically, the ability to reduce disc stress and posterior annulus bulging depends upon the flexible spring cord and expandable spacer (2 mm longer after distraction). The spacer length is thought to play a key role during extension and lateral bending, stabilizing the adjacent and transition segments with minimal effect in rotation. Liu et al. [25] showed that an increase in Dynesys cord pretension resulted in an increase in flexion stiffness from 19.0 to 64.5 Nm/deg, with a prominent increase in facet contact force of 35% in extension and 32% in torsion. Also found was a significant increase in stress applied onto the pedicle screws in flexion and lateral bending. Thus, the trade-off of lower cord pretension might afford higher mobility in sharing the loading force, to lower the pedicle screw stress in flexion and minimize the facet contact force in extension [26]. A similar concern about excess cord pretension was expressed by Ferraro et al. [27] in a 2020 study. In the DTO system, the screw-spacer linkage acts to balance the application of the vertebral loading force and cord pretension at the two sides. Chien et al. [28] revealed that the Dynesys screw-spacer supplies only 33% of the contact force; the other 67% of load comes from cord pretension, muscular contractions, and the body weight still borne by the vertebrae when the cord was extended to 300 N. As a result, excessive shearing loads in unstable spondylolisthesis accompanied by contact stress imposed on the screw-spacer linkage subsequently lead to material fatigue under a heavy vertebral load and extreme flexion. This phenomenon might be a reasonable explanation for the implant loosening in the trauma case.

According to the ASD rates reported in the literature, Kashkoush et al. [29] reported a promising reduction in the subsequent development of adjacent-segment disease with his 10 years’ experience with DTO instrumentation. However, Herren et al. [22] showed comparable rates of radiologically detectable ASD (28.6% for PLIF vs. 26.7% for topping-off, total 29 patients). In a 2018 study by Kuo et al. [30], neither postoperative antero- nor retro-listhesis (15.2% in dynamic fusion vs. 17.4% in PLIF, *p* = 0.92) nor endplate degeneration evaluated with Modic classification (1.8% in PLIF vs. 6.5% in dynamic fusion, *p* = 0.30) could show statistical significance. One reason for the lack of significance in reducing the ASD rate in this study might be that all patients had a certain degenerative alteration in the disc adjacent to the instrumentation level. In other words, it might be more clinically important to focus on how to delay ASD rather than prevent it. As mentioned above, existing degenerative changes might play a prominent role in the development of ASD. As a result, we should consider degeneration as an ongoing process rather than stages with definite cut points or thresholds. If the incidence rate of ASD is the only parameter analyzed, the existing degenerative change will become a confounding factor. To correct this bias, we additionally analyzed data for the L2–L3 segment. In our hypothesis, the L2–L3 segment represents the non-instrumented segment which is relatively “intact” or “neutral.” In the present study, degenerative change in the L3–L4 segment, including both disc height reduction and listhesis distance, showed a minor increase compared to the L2–L3 segment. In our opinion, this result may indicate that this dynamic stabilization technique can decelerate the degenerative process. Similar favorable findings in motion angular change were noted in the L3–L4 segment as well.

In terms of the surgical technique, some studies used total laminectomy when discussing hybrid instrumentation techniques [25,31]. In our opinion, the better surgical decompression strategy is bilateral laminotomies, which avoid the destruction of the spinous process and posterior ligamentous complex. To prevent postoperative adjacent segmental disease, the posterior complex should be preserved as much as possible during surgery [32]. Iorio et al. [33] also proposed that both the interspinous and supraspinous ligaments contribute greatly to spinal stability by providing resistance to flexion via a long moment arm from the spinous process to the instantaneous axis of rotation. Thus, the destruction of the posterior complex during the operation may be a potential confounding factor related to postoperative ASD.

Biomechanical and clinical studies have suggested that the increased stress under different loading conditions (flexion, extension, lateral bending, axial rotation) [34] and the change in range of motion [35] at the upper adjacent level after rigid fixation may lead to the risk of ASD at the index level. In our study, disc height and listhesis distance revealed that ASD change at the index level was delayed and also minimal. This phenomenon might be attributed to the shear-load effect on the dynamic instrumentation, which shifted the tensile and compression forces to the upper rigid transition screw in the construct. In addition, we also noted less angular change at the index level while in motion.

There are some limitations to this study. The sample size in this study was small, enrolling only 31 patients in the final analysis due to our strict inclusion and exclusion criteria. Second, the last follow-up period was set at 24 months after the operation. Observation for a longer period may have shown an increased effect of dynamic instrumentation in delaying ASD. Lastly, the lack of clinical outcomes and postoperative magnetic resonance imaging follow up are both limitations to this study.

## 5. Conclusions

Our study on the use of hybrid dynamic stabilization with the DTO system in lumbar spinal stenosis patients with spondylolisthesis reveals that the L3–L4 segment, targeted by our intervention, experienced less disc height reduction, a delayed increase in listhesis distance, and a slower onset of ASD compared to the L2–L3 and L5–S1 segments. Additionally, a significant reduction in angular motion change at the L3–L4 level suggests a stabilizing effect of the DTO system. While our findings indicate a delay in the progression of ASD rather than its outright prevention, they highlight the potential of this technique in minimizing degenerative changes in the treated segment. Despite limitations, such as a small sample size and a two-year follow-up period, our results suggest that hybrid dynamic stabilization may be a promising strategy for delaying ASD in this patient population, warranting further investigation with larger cohorts and extended follow up.

## Figures and Tables

**Table 1:** Demographic characteristics of the study cohort (*n* = 31).

Characteristic	Value
**Mean Age (years)**	68.5 ± 7.5
**Gender**	
Male	20 (64.5)
Female	11 (35.5)

Values are presented as number (%) or mean ± standard deviation.

**Table 2:** Preoperative and 2-year postoperative follow-up radiographic data for various segments.

Location	Preoperative		2-Year Postoperative		*p* Value
	mean	±SD	mean	±SD	
**L2–L3 segment**					
**Average disc height (cm)**	0.74	±0.22	0.48	±0.20	<0.001
Anterior disc height	0.86	±0.33	0.60	±0.27	<0.001
Posterior disc height	0.62	±0.23	0.37	±0.16	<0.001
**Listhesis distance (cm)**	0.24	±0.09	0.51	±0.12	<0.001
**Angular motion change** **(degrees)**	5.46	±3.67	5.18	±3.22	0.943
**L3–L4 segment**					
**Average disc height (cm)**	0.75	±0.26	0.52	±0.20	<0.001
Anterior disc height	0.91	±0.33	0.65	±0.27	<0.001
Posterior disc height	0.58	±0.23	0.39	±0.19	<0.001
**Listhesis distance (cm)**	0.25	±0.09	0.43	±0.09	<0.001
**Angular motion change**	6.58	±3.78	4.34	±3.29	0.020
**L5–S1 segment**					
**Average disc height (cm)**	0.85	±0.28	0.56	±0.21	<0.001
Anterior disc height	1.04	±0.37	0.72	±0.29	<0.001
Posterior disc height	0.65	±0.26	0.40	±0.18	<0.001
**Listhesis distance (cm)**	0.28	±0.08	0.48	±0.12	<0.001
**Angular motion change**	7.90	±3.92	8.19	±3.38	0.096

SD: standard deviation.

## Data Availability

Data are available upon reasonable request. The datasets used during the current study are available from the Taichung Veterans General Hospital; however, restrictions apply regarding the availability of these data, as they are not publicly available. However, the data are available from the corresponding author upon reasonable request and with permission from the Taichung Veterans General Hospital.

## Abbreviations

ASD: adjacent segment disease; ADH: anterior disc height; AP: anteroposterior; DTO: Dynesys-Transition-Optima; MRI: magnetic resonance imaging; NA: not applicable; PDH: posterior disc height; PLIF: posterior lumbar interbody fusion; ROM: range of motion; TLIF: transforaminal lumbar interbody fusion; SD: standard deviation; Yrs: years.
